# Centrifuge-free cell radiolabeling using acoustophoresis

**DOI:** 10.1038/s41598-025-09670-z

**Published:** 2025-07-08

**Authors:** Stephen S. Adler, Emma Stevenson, Julia Alsveds, Peter L. Choyke, Noriko Sato

**Affiliations:** 1 Clincal Research Directorate, Frederick National Laboratory for Cancer Research, Fredrick, MD USA; 2 University of Alabama at Birmingham, Birmingham, USA; 3 National Cancer Institute, Bethesda, MD USA; 4 AcouSort AB, Lund, Sweden; 5 9000 Rockville Pike, Building 10, Room B3B51, Bethesda, MD 20892 USA

**Keywords:** Cell radiolabeling, Acoustophoresis, ^89^Zr-oxine, Cell washing, Cell up-concentration, Biomedical engineering, Cancer imaging

## Abstract

Tracking ex vivo radiolabeled cells using radionuclide imaging such as positron emission tomography is an emerging method for evaluating cell-based therapies. Traditional radiolabeling requires a centrifuge in multiple steps to optimize labeling and remove unbound radiotracers. With the goal of automating the radiolabeling procedure, we explored an acoustophoresis-based approach for radiolabeling cells, eliminating the need of using a centrifuge, simplifying the design of this future device. The AcouWash 2 (AcouSort AB, Lund, Sweden), an acoustophoresis based cell washing device, was evaluated for its ability to label EL4 murine T lymphoblasts with zirconium-89 (^89^Zr)-oxine without centrifugation. The AcouWash 2 successfully replaced the culture medium with a protein-free buffer, as required for ^89^Zr-oxine cell labeling. Additionally, it was able to concentrate EL4 cells by a factor of 5.6 ± 0.4, achieving or exceeding the optimal labeling cell density. After cell incubation with ^89^Zr-oxine, AcouWash 2 exchanged the incubation solution with a solution for infusion, removing unbound ^89^Zr-oxine. These steps eliminated the need for centrifugation at each stage of the labeling procedure. The resulting radiolabeled EL4 cells exhibited labeling metrics, specific activity, percent labeling efficiency, percent free ^89^Zr-oxine in the suspension buffer and cell viability, comparable to those obtained from conventional centrifuge-based method. Our results demonstrate that the radiolabeling can be performed entirely using acoustophoresis, which paves the way for developing a fully automated radiolabeling device based on acoustophoresis technology.

## Introduction

Immune cell therapy has been performed to treat a growing list of cancer types and other diseases^1^. However, once immune cells are transferred to the recipient, current monitoring methods are limited to measuring the therapeutic outcome. Information such as how many of the infused cells migrate to the target, whether cell engineering increase or decrease homing, and whether therapeutic efficacy depends on trafficking of the cells to the target would help in developing next generation cell therapies. One method to monitor the therapy is to label a fraction of the infused cells with a radioisotope which enables imaging and tracking of the cell distribution within the patient receiving the treatment^2–4^.

Key to imaging the cells is the radiolabeling process by which one attaches an imaging radionuclide ligand to the cells. Currently, it is performed as a multi-step manual procedure^5,6^. This requires a lab person to carefully count the cells and centrifuge them in sequential steps. This includes washing the cells in different buffers and preparing them at a specific concentration for the radiolabel incubation. Labeled cells are then washed again by centrifugation multiple times to finally obtain the cells ready for infusion into a patient. The work involves careful handling of the cells and exposure to radioactivity.

The goal of our research is to develop an automated system for immune cell radiolabeling. It would reduce the number of manual steps currently required to perform the radiolabeling, reduce human error, and would be safer for lab workers. Such a system must be designed to be placed in Current Good Manufacturing Practices (cGMP) radio-pharmacies where the radiolabeling procedure would be performed. Although centrifugation is the present main method of washing and concentrating the cells used during the cell radiolabeling process, designing an automated radiolabeling system using a centrifuge would require a complex robotic design to place and remove the vials and possibly make the system too large to fit on top of a bench in a cGMP lab. A successful design would use a technology based on miniaturization enabling the system to have a compact form factor.

The field of microfluidics has developed technologies which address the challenges of performing cell-based procedures at small scales. A subfield of microfluidics called acoustophoresis^7^ uses pressure forces within laminar flows caused by ultrasound transducers to manipulate cells suspended in fluids in unique ways^8^. Methods have been developed which allow capturing cells within laminar flows as well as moving cells between buffer solutions in effect providing a method of washing cells^9–13^. Various applications of acoustophoresis have been developed to date such as the assembly of organotypic cell clusters^14,15^ and nanoparticle enrichment and separation^16^.

Microfluidics, and its subfield of acoustophoresis, provide the solution to designing the desired small form factor, standalone integrated system for radiolabeling cells. Because our goal is to design an automated system for immune cell radiolabeling using acoustophoresis technology, we must first validate that using acoustophoresis to radiolabel cells can be done as effectivity as the current methods using a centrifuge. The work we report here is the validation work showing that one can use acoustophoresis as a core technology to radiolabel immune cells.

This study is a continuation of our effort to validate the use of acoustophoresis to perform cell radiolabeling. Previously^17^, we used an acoustophoresis cell washing system, the AcouWash (AcouSort, Lund, Sweden)^18^ and validated its ability for replacing the centrifuge in the radiolabeling procedure for all the cell washing steps. These includes replacing the culture medium to a buffer solution suitable for labeling cells. And then after labeling cells with zircounim-89 (^89^Zr)-oxine, an ex vivo cell labeling agent for positron emission tomography (PET) imaging, it was used to wash off unbound ^89^Zr-oxine. The comparison between the cells which underwent the AcouWash radiolabeling method and the centrifuge method showed comparable levels of radiolabeling success in the percent labeling efficiency, radiolabeled specific activity, and percent cell recovery. Importantly, acoustophoresis process using the AcouWash system did not hinder cellular viability and activation function measured by interferon gamma production function when in murine T cells. However, a key step of preparing cells at the proper concentration for radiolabeling was left out of the validation work. The acoustophoresis cell washing technology built into the AcouWash system did not have the ability to substantially increase the cell density to the required concentration levels for the incubation step where the ^89^Zr-oxine labeling occurs. Therefore, a centrifuge was required for this step and we were not able to fully achieve centrifuge-free radiolabeling.

With the advances in acoustophoresis technology, AcouSort released their next generation acoustophoresis cell washing system, the AcouWash 2. This new version made advances to their buffer flow control system achieving higher flow rates by a factor of 5. This in turn allows one to operate the system with larger initial to final flow rate differences compared to the original AcouWash system, achieving the cell up-concentration capability required by the radiolabeling procedure.

In this study, we used the AcouWash 2 to achieve the final goal of performing centrifuge-free cell radiolabeling. With the expanded set of operational parameters of the AcouWash 2, a series of tests were conducted to understand its performance, before using it for cell radiolabeling. This included tuning the wash buffer density and the flow settings to achieve maximum up-concentration. After establishing the optimized acoustophoresis cell washing protocol, the radiolabeling procedure was reevaluated against the centrifuge-based procedure by comparing labeled specific activity, percent labeling efficiency, percent free ^89^Zr-oxine, cell recovery, and cell viability. Herein we report the results of those tests and demonstrate that the AcouWash 2 can replace the centrifuge in both the cell washing and the cell up-concentration steps.

## Materials and methods

### Acoustophoresis cell washing

**Fig. 1:**
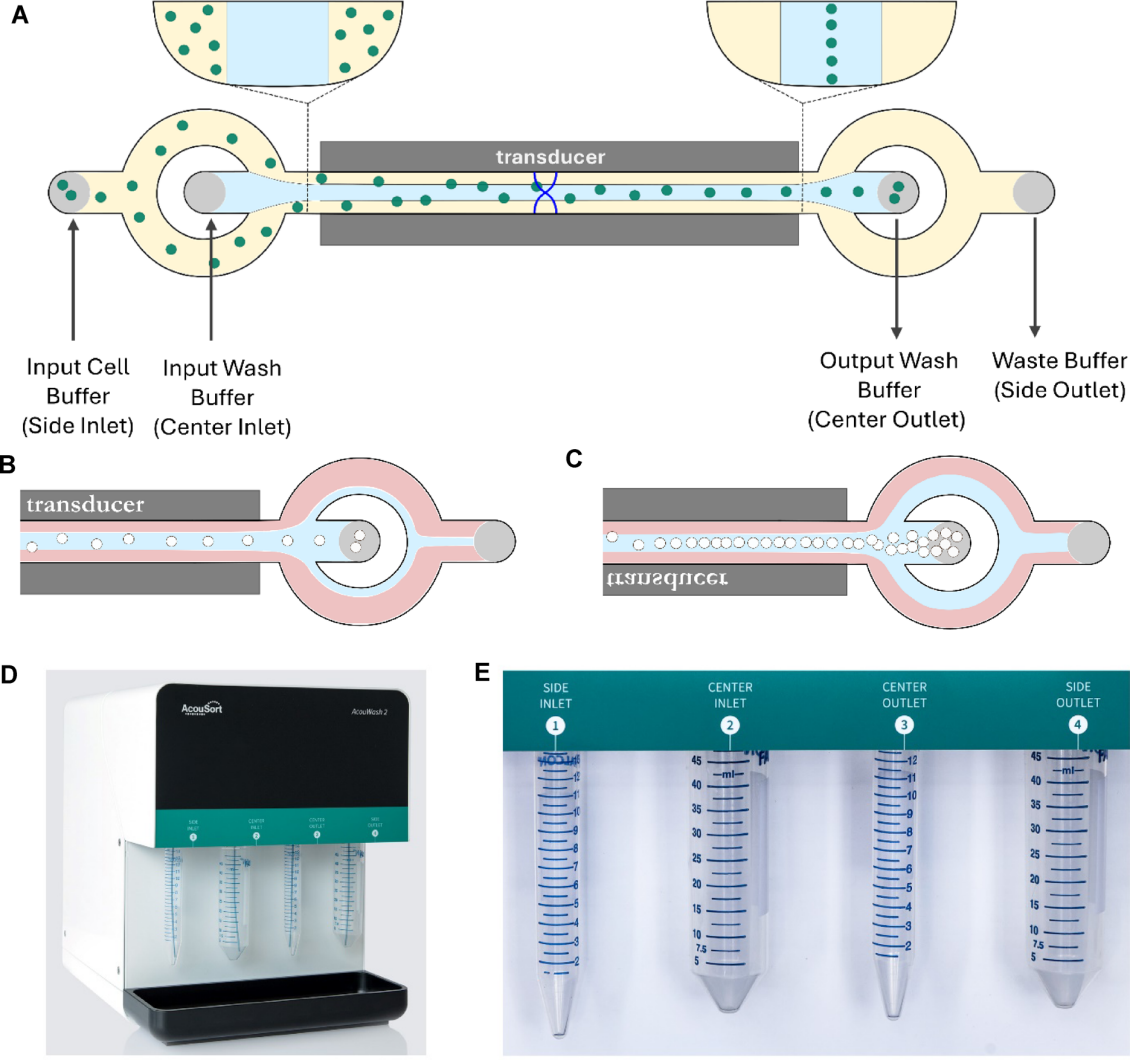
The acoustophoresis cell washing process works by having two buffers flow through a micro channel in which a standing acoustic field is induced by an ultrasound transducer. The acoustic force acts on the cells in the channel, focusing them at the node of the standing acoustic wave at the center of the channel (**A**). Using different flow rate settings for the input and output cell buffers, a simple cell washing step is completed in which the cell concentration remains constant (**B**) or change in the cell density by up-concentrating the cells going into the outlet (**C**). For this work, an AcouWash 2 was used (**D**). The cells are attached to the side inlet, the wash buffer is attached to the center inlet, the washed cells come out of the center outlet and the waste goes into the side outlet (**E**)

The acoustophoresis cell washing process works by channeling two buffer solutions through a microchannel (Fig. 1). The two buffers enter the microchannel through separate inlets. The buffers enter the microchannel such that three streams of liquids in a laminar flow are established. One buffer feeds the two-outside streams, and the other buffer feeds the middle one as they flow through the microchannel (Fig. 1A). The first buffer is referred to in this work as the “input cell buffer” containing the cells being washed. The second buffer is referred to as the “wash buffer” into which the cells are transferred. An ultrasound transducer is placed underneath the microchannel and is actuated at a resonance frequency to create a standing wave between the channel walls. This creates a pressure field within the microchannel, which causes the cells to move from the two outside laminar flow streams focusing them into the pressure node at the center of the channel. The two buffers exit the microchannel through two outlets with the wash buffer exiting the first outlet with the transferred cells and a mix of the original buffer and wash buffer exiting via the second outlet. By controlling the flow rates between the input and output wash buffers, one can control the volume of wash buffer exiting through the two outlets, thus controlling the final concentration of cells in the wash buffer (Fig. 1B,C).

For the two buffers to flow through the acoustic field while maintaining their laminar stream equilibrium, the buffer in the stream flowing through the center needs to have a higher density than the second buffer flowing along the two the side streams. If not, a relocation of the two buffers occurs, spoiling the washing efficiency of the system^19^. Therefore, the wash buffer must have a higher density than the initial cell suspension buffer for the acoustophoresis washing to be effective. Otherwise, the output wash buffer will be contaminated with the original cell suspension buffer.

The AcouWash 2 (AcouSort, Lund, Sweden) was used for this work (Fig. 1D). It requires the use of two 15 and two 50 mL tubes (Fig. 1E). These tubes are fastened to two inlets and two outlets named side inlet (15 mL tube), center inlet (50 mL tube), center outlet (15 mL tube) and side outlet (50 mL tube). These correspond to the input cell buffer, input wash buffer, output wash buffer and waste buffer, respectively.

The cells being washed are placed in a 15 mL tube and fastened to the side inlet. The wash buffer is placed in a 50 mL tube and fastened to the center inlet. A 15 mL tube and 50 mL tube are fastened onto the center and side outlets. The center outlet tube will receive the cells suspended in the output wash buffer while the waste will end up in the side outlet tube.

To perform a cell wash with the AcouWash 2, one needs to set the flow rates of the two buffer solutions coming in through the side and center inlets and exiting through the center and waste outlets. Through the user interface controlling the AcouWash 2, one sets the flow rates for the side inlet, center inlet, and center outlet. The side outlet flow rate is unrestrained and will automatically adjust to ensure the total output flow rate equals the total input flow rate.

Flow rate settings are set using the notation (side inlet flow rate)/(center inlet flow rate) (center outlet flow rate)/(side outlet flow rate). As an example, a flow rate setting of 100/200 100/200 means that the flow in the side inlet is 100 µL/min, 200 µL/min into the center inlet, 100 µL/min flow out the center outlet, and 200 µL/min flow out of the side outlet. Because the center inlet buffer flows through the microchannel connecting to the center outlet and the center inlet has twice the flow rate as does the center outlet, the excess flows into the waste.

The flow rates to the side inlet, center inlet and center outlet can be set within the range of 100 µL/min to 900 µL/min. Therefore, there are several flow configurations which affect the operation of the wash cycle. Flow settings of the form X/Y X/Y are good for washing without increasing the concentration of the cells (Fig. 1B). Flow settings of the form X/Y Y/X change the concentration of the cells such that the resulting up-concentration factor is X/Y (Fig. 1C).

### EL4 cells

Murine T lymphoblast EL4 cells were purchased from the American Type Culture Collection and were cultured in RPMI 1640 medium (Thermo Fisher Scientific) supplemented with 10% fetal Bovine Serum, Value, heat inactivated (FCS, Thermo Fisher Scientific), 100 IU/ml penicillin/100 µg/ml streptomycin (Thermo Fisher Scientific), and 50 µM 2-mercaptoethanol (Sigma-Aldrich), referred to culture medium hereafter.

### Wash buffer density determination

As mentioned, for the acoustophoresis washing to work effectively, the wash buffer needs to have a higher density than the input cell suspension buffer to avoid buffer mixing in the wash process. In our case, the input cell suspension buffer is the culture medium, and the wash buffer is phosphate buffered saline (PBS). Since the cell culture medium has a higher density than PBS, a special heavy density PBS (H-PBS) needs to be prepared for it to have a higher density than culture medium. To do this, a small amount of Lymphocyte Separation Medium (Lonza) was added to the PBS which increases its density. PBS and Lymphocyte Separation Medium have densities of 1.008 g/ml and 1.077 g/ml, respectively.

Eight different H-PBS solutions were prepared with varying densities by adding 1 part per 10, 15, 20, 25, 30, 50 and 100 volume ratios of Lymphocyte Separation Medium to PBS with the final sample solution being pure PBS with no Lymphocyte Separation Medium added. This yielded eight different H-PBS solutions with densities of 1.0140, 1.0120, 1.0110, 1.0104, 1.0099, 1.0091, 1.0084 and 1.0077 g/ml, respectively.

Using these H-PBS solutions, the AcouWash 2 was configured with 4 different wash flow settings of 100/200 100/200, 200/400 200/400, 300/600 300/600 and 450/900 450/900 µL/min and washes were performed for each flow setting and H-PBS density solution with the resulting medium mixing measured.

To measure the magnitude of buffer mixing, 10–30 kBq of ^89^ZrCl_4_ (generated in house) was added to the culture medium before starting the wash. After the wash finished, the output wash and waste volumes were measured gravimetrically. From each output buffer, 0.5 ml samples were extracted and the radioactivity levels measured with a microdose calibrator^20^. The total radioactivity $A$ of each output buffer was then calculated using the equation

1 $$A=\frac{{A}_{s}}{.5}V$$

where ${A}_{s}$ is the activity in the 0.5 mL sample, and $V$ is the volume of the output buffer. The mixing ratio $R$ was measured using the equation

2 $$R=\frac{{A}_{O}}{{A}_{O}+{A}_{W}}$$

where ${A}_{O}$ is the total activity estimated from the output wash buffer and ${A}_{W}$ is the total activity estimated from the waste sample.

### Cell concentration measurements

Central to this work is the ability to wash cells while increasing their concentration at the end of the wash cycle. We define the cell up-concentration factor $U$ as the ratio of the cell density after the wash cycle, ${D}_{A}$, to the cell density before the wash cycle, ${D}_{B}$.

3 $$U={D}_{A}/{D}_{B}$$

A theoretical maximum cell up-concentration factor $U$ can be calculated as

4 $$U={F}_{I}/{F}_{O}$$

where ${F}_{I}$ is the side inlet flow rate and ${F}_{O}$ is the center outlet flow rate.

A set of wash cycles were performed to measure the cell up-concentration factor from the measured flow rates through the inlets and outlets of the AcouWash 2. This was done for the flow rate settings of 300/100 100/300–900/100 100/900 in increments of 100.

The input tube was filled with 18.6 ml of 20:1 H-PBS containing EL4 cells at a density of 1 × 10^6^ cells/mL. The wash buffer input tube was prepared with 15 ml of 20:1 H-PBS. The flow rate settings for the first wash cycle were set to 900/100 100/900. A short wash cycle was performed to flush out excess PBS in the output tube henceforth denoted as a “flush cycle”. A second wash cycle was then performed maximizing the cell concentration in the output cell sample. The measured flow rate concentration factor $U$ was measured using gravimetrically derived volumes of the input and output cell suspensions with the equation

5 $$U=\frac{{V}_{I}}{{V}_{f}+{V}_{O}}$$

where ${V}_{I}$ is the volume of the input cell buffer sample for both the flush and wash cycles, ${V}_{f}$ is the output volume of the flush cycle and ${V}_{O}$ is the output volume after the wash cycle. The cell density was measured in the output washed cell suspension buffer and the cell concentration was then calculated using Eq. (3).

Replacing the output cell buffer tube with a new one, the next wash cycle was performed at 800/100 100/800 collecting the same data of input and output wash volumes and output cell density. This was repeated for flow rate settings of 700/100 100/700, down to 300/100 100/300 decrementing the side inlet flow rate by 100 µL/min for each new wash cycle.

### Cell up-concentration as a function of input cell concentration

The maximum effective cell up-concentration settings of 600/100 100/600 were used to measure the cell up-concentration as a function of the input cell concentration. Throughout the many radiolabeling tests, the cell density was measured before and after the up-concentration wash, in the step which prepares the cells for incubation with ^89^Zr-oxine. The measured up-concentration factors are used to determine the limits of absolute cell up-concentration.

### Cell radiolabeling method

**Fig. 2:**
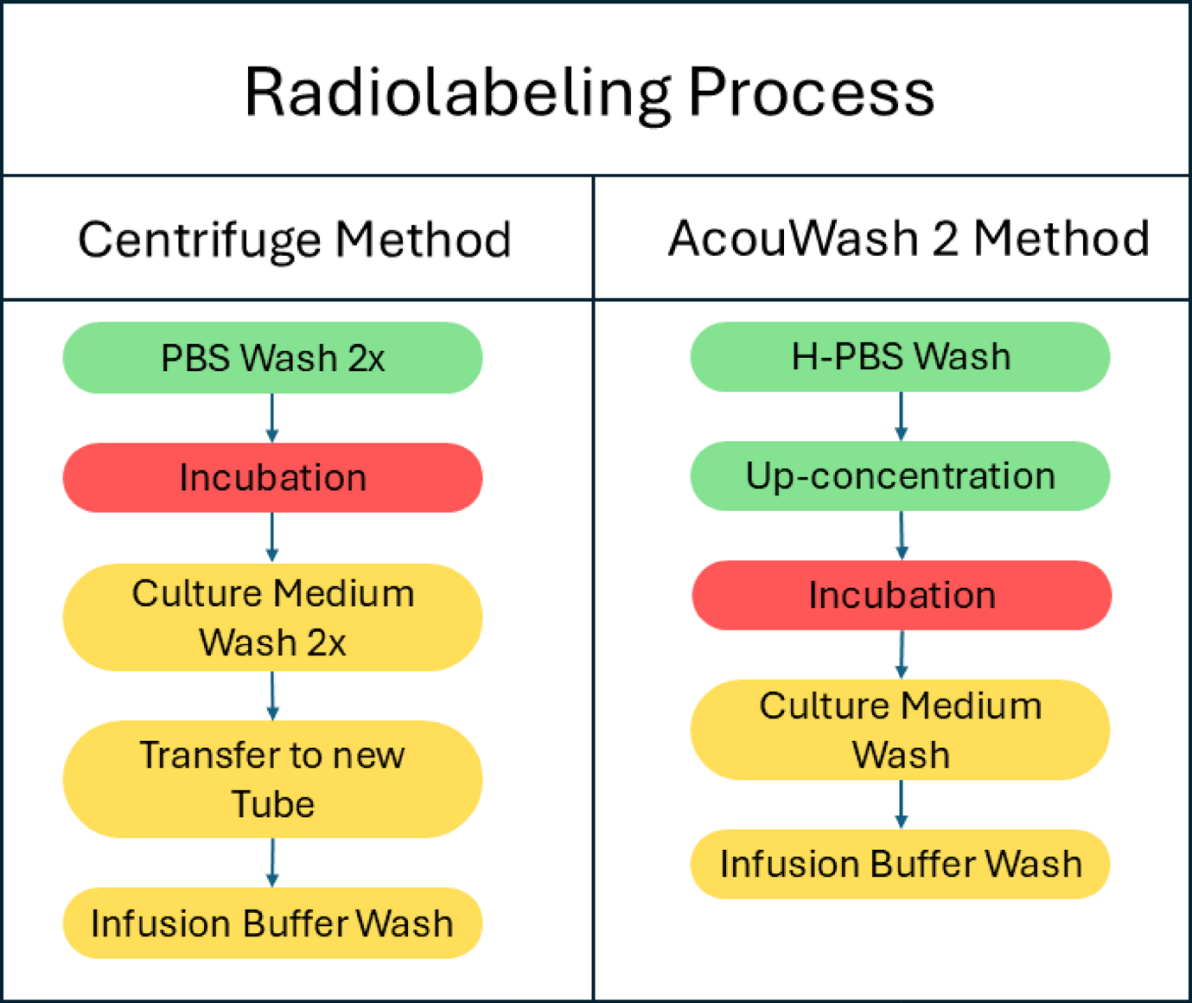
The radiolabeling procedure is summarized by grouping the steps in three different categories. In green are the pre-incubation washing steps, in red the incubation step, and in yellow the post incubation steps. For this work, the two procedures were run in parallel starting with the same unlabeled cells in culture medium. This was done to minimize any differences which could arise from having the cells sit in suspension while one of the two procedures was being done, affecting the initial condition of the cells which could result in different radiolabeling results. It takes about 2 h to run through the full cell labeling procedure, including all the cell counting which occurs at each labeling step.

The radiolabeling procedure can be broken down into three parts (Fig. 2). The first part are the steps needed to harvest the cells from culture flasks and prepare them in a PBS buffer free of the culture medium in which the cells are grown. The second part radiolabels the cells. In this part, cells are prepared at a specific volume and density where ^89^Zr-oxine is added and incubated for 15 min. The third part is the post radiolabeling washing procedure in which the labeled cells are washed with protein-containing buffer to remove any remaining unbound ^89^Zr-oxine. The radiolabeled cells are then suspended in the final infusion buffer. ^89^ZrCl_4_ and ^89^Zr-oxine were generated in-house according to the methods previously reported^5,6^.

### AcouWash 2 cell labeling procedure

With the operational parameters set for using the AcouWash 2 in the cell radiolabeling procedure, the following are the steps to radiolabel EL4 cells.

H-PBS was prepared using a 20:1 PBS to Lymphocyte Separation Medium mixture. This H-PBS will be used throughout the labeling process when cells require washing in H-PBS.

The infusion buffer was prepared using Plasma-Lyte A (Baxter) with 4% Albumin Bovine Fraction V (BSA, MP Biomedicals). This is the buffer where the radiolabeled EL4 cells will be suspended at the end of the radiolabeling procedure.

The AcouWash 2 was operated in two different modes. The first was the wash mode where the flow settings were set to 400/800 400/800 µL/min. The cell concentration remained constant using these flow settings. The second flow setting was the up-concentration mode in which the flow settings were set to 600/100 100/600 µL/min.

EL4 cells from culture were suspended in 10 to 20 mL of culture medium (initial cells) and were counted using a Luna FX7 cell counter (Logs Biosystems, Anyang, South Korea) recording the cell density and viability. The cell viability was measured using a Trypan blue stain (0.4%). The starting concentration ranged from 4 × 10^6^ to 10 × 10^6^ cells/mL.

A volume of 2 mL of the cells suspended in culture medium was transferred into a 15 mL tube. The volume was measured gravimetrically and recorded, after which the tube was screwed into the side inlet of the AcouWash 2.

The first wash was performed with the AcouWash 2 set to wash mode in which the cells in culture medium were washed with H-PBS. The cells were counted post wash to record the cell density and viability.

The cells suspended in H-PBS were up-concentrated using H-PBS as the wash buffer. The up-concentrated output buffer volume was measured gravimetrically, and the cells were counted to record their density and viability. The total number of cells was calculated from the volume and density.

The equation for the required incubation cell density ${D}_{inc}$ is

6 $${D}_{inc}=\frac{{N}_{cell}}{{V}_{inc}}=\frac{1000\,{E}_{lab}{S}_{\text{ZrOx}}}{{{F}_{PBS}S}_{lab}}$$

where ${D}_{inc}$ is the incubation density in units of 10^6^ cells/mL, ${N}_{cell}$ is the number of cells in units of 10^6^ cells, ${V}_{inc}$ is the incubation volume in units of µL, ${E}_{lab}$ is the labeling efficiency, ${F}_{PBS}$ is the volume factor of PBS to ^89^Zr-oxine as in ${F}_{PBS}$ parts PBS to 1 part ^89^Zr-oxine, ${S}_{\text{ZrOx}}$ is the ^89^Zr-oxine specific activity in units of kBq/µL and ${S}_{lab}$is the labeling specific activity in units of kBq/10^6^ cells. Equation (6) is expressed in this form because the four variables ${E}_{lab}$, ${S}_{\text{ZrOx}}$, ${F}_{PBS}$, and ${S}_{lab}$ are the operational parameters which govern the incubation process of cell radiolabeling procedure. One should note that the labeling efficiency, ${E}_{lab}$, although has a variable outcome depending on cell handling among other issues, it is treated as a constant and set to 30%. This 30% value was determined as the average labeling efficiency for the centrifuge based method when ^89^Zr-oxine was first developed as the imaging ligand for immune cell therapies^5^.

The incubation volume can be derived from Eq. (6) as

7 $${V}_{inc}=\frac{{N}_{cell}{F}_{PBS}{S}_{lab}}{1000\,{E}_{lab}{S}_{ZrOx}}$$

After the up-concentration wash step, the volume of the cell suspension was measured. The cell density of the concentrated cell suspension should be higher than the incubation cell density calculated using Eq. (6). If this was the case, a volume of H-PBS was added to the up-concentrated cell suspension to equal the incubation volume ${V}_{inc}$. Finally, a volume equal to ${V}_{inc}/{F}_{PBS}$ of ^89^Zr-oxine was added to the suspension, the mixture was vortexed and incubated for 15 min at room temperature. During the incubation, the ^89^Zr-oxine activity was measured using a microdose calibrator.

At the end of the 15-minute incubation, 1 mL of culture medium was added to the incubation vial, which halted the ^89^Zr-oxine binding. The suspension was washed once with culture medium followed by a wash with the infusion buffer Plasma-Lyte A with 4% BSA. This sample was the one which would be infused into a patient. A cell count was done with a sample of this suspension buffer to measure the cell density and viability. The volume of the sample was measured gravimetrically and used to calculate the total number of cells.

The fractional free ^89^Zr-oxine in the supernatant was then measured as described in the Radiolabeling Assessment subsection below.

### Centrifuge cell labeling procedure

A 1 mL initial cell suspension in culture medium was transferred to a 1.5 mL centrifuge vial. The cells were washed twice with PBS using a microcentrifuge at 1500×g for 3 min. After the second wash, the suspension volume was measured gravimetrically, and the cells were counted, recording the cell density and viability. The total number of cells was calculated from the density and volume of the sample. Using Eq. (7), the incubation volume was calculated, and the cells were spun down and resuspended with ${V}_{inc}$ µL of PBS. A volume of ${V}_{inc}/{F}_{PBS}$ µL of ^89^Zr-oxine was added to the incubation volume and vortexed. The incubation sample was counted, the volume gravimetrically measured, and the activity was measured in the microdose calibrator.

The cells were incubated for 15 min at room temperature after which 1 mL of culture medium was added to halt the incubation process. The cells were washed with culture medium twice. The cells were resuspended in Plasma-Lyte A with 4% BSA, transferred to a new 1.5 mL vial and spun one last time. The cells were resuspended in Plasma-Lyte A with 4% BSA, the suspension volume was measured gravimetrically, the cells were counted, and the activity was measured in the microdose calibrator.

The fractional free ^89^Zr-oxine in the supernatant was then measured as described below.

### Radiolabeling assessment

After the radiolabeling procedures, the labeled specific activity, percent labeling efficiency, cell recovery, percent free ^89^Zr-oxine and cell viability were determined.

At the end of the labeling procedures, the suspension volume was measured gravimetrically, the cells were counted, and the activity was measured using a microdose calibrator. The cell counter reports the cell density, therefore the ***labeled specific activity***, ${S}_{lab}$, was calculated using the equation

8 $${S}_{lab}=\frac{{A}_{ZrOx}}{{D}_{cell}{V}_{cell}}$$

where ${A}_{ZrOx}$ is the ^89^Zr-oxine activity of the suspension in kBq, ${D}_{cell}$ is the density of cells in the suspension in units of 10^6^ cells/mL, and ${V}_{cell}$ is the suspension volume in mL.

The ***percent labeling efficiency***, ${E}_{lab}$, was calculated using the equation

9 $${E}_{lab}=\frac{{S}_{lab}}{{S}_{inc}}100\%$$

where ${S}_{lab}$ and ${S}_{inc}$ are the labeled and incubation specific activities respectively.

The ***percent cell recovery*** is the percent ratio of the labeled cell count to the initial cell count and is a measure of how many cells are lost through the radiolabeling procedure.

The ***percent free*** ^***89***^***Zr-oxine*** measures the amount of unbound ^89^Zr-oxine left in the supernatant at the end of the radiolabeling procedure and is an indication of how well the excess ^89^Zr-oxine is removed from the suspension buffer. To measure this, the final labeled sample was spun down using a centrifuge (at 1500×g for 3 min) and 0.5 mL sample of supernatant was pipetted into a separate vial. The ^89^Zr-oxine activity of this sample was measured in the microdose calibrator. The precent free ^89^Zr-oxine activity, $F$, was calculated using the following equation

10 $$F=\frac{\frac{{A}_{ZrOx}}{.5}{V}_{inf}}{{A}_{lab}}100\%$$

where ${A}_{ZrOx}$ is the ^89^Zr-oxine activity of the 0.5 mL supernatant sample, ${V}_{inf}$ is the volume of the infusion buffer, and ${A}_{lab}$ is the total activity of the radiolabeled cell sample.

The ***cell viability*** was measured by flow cytometry analysis of the cells stained with Annexin V and propidium iodide (PI) as described below. The cell viability was measured at the beginning and at the end of the radiolabeling procedure.

### Flowcytometry analysis

The cells were suspended in 1x Binding Buffer for annexin V staining (Clontech, discontinued), and stained with Annexin V-FITC (AV, BioLegend) and PI (BD Pharmingen) for 15 min at room temperature. The cells were washed and resuspended in the annexin V binding buffer. The stained cells were applied to a CytoFLEX flow cytometer (Beckman Coulter). The acquired data were analyzed using FlowJo software v10.0.0 (BD Biosciences, flowjo.com) to determine the percent cell sample which was healthy (AV-PI-), apoptotic (AV + PI-), or necrotic (PI+) for the initial cell sample pre radiolabeling, the centrifuge radiolabeled cell sample and the AcouWash radiolabeled cell sample.

### Statistical analysis

GraphPad Prism 10.1.1 (www.graphpad.com) was used to perform statistical analyses. Wilcoxon matched-pairs signed rank test were performed to compare the results of the centrifuge versus AcouWash 2 radiolabeling test measurements. These include labeled specific activity, percent labeling efficiency, percent free ^89^Zr-oxine and cell recovery.

One-way analysis of variance (ANOVA) was used to compare the flow cytometry cell viability results. It was used to determine if there were any significant differences in the cell viability between the original cell sample, the centrifuge radiolabeled cell sample, and the AcouWash 2 radiolabeled. The three cell viability categories of healthy, apoptotic and necrotic were compared independently.

P values less than 0.05 were considered significant. The data are shown as mean ± standard deviation.

## Results

### AcouWash 2 performance and operational parameters

**Fig. 3:**
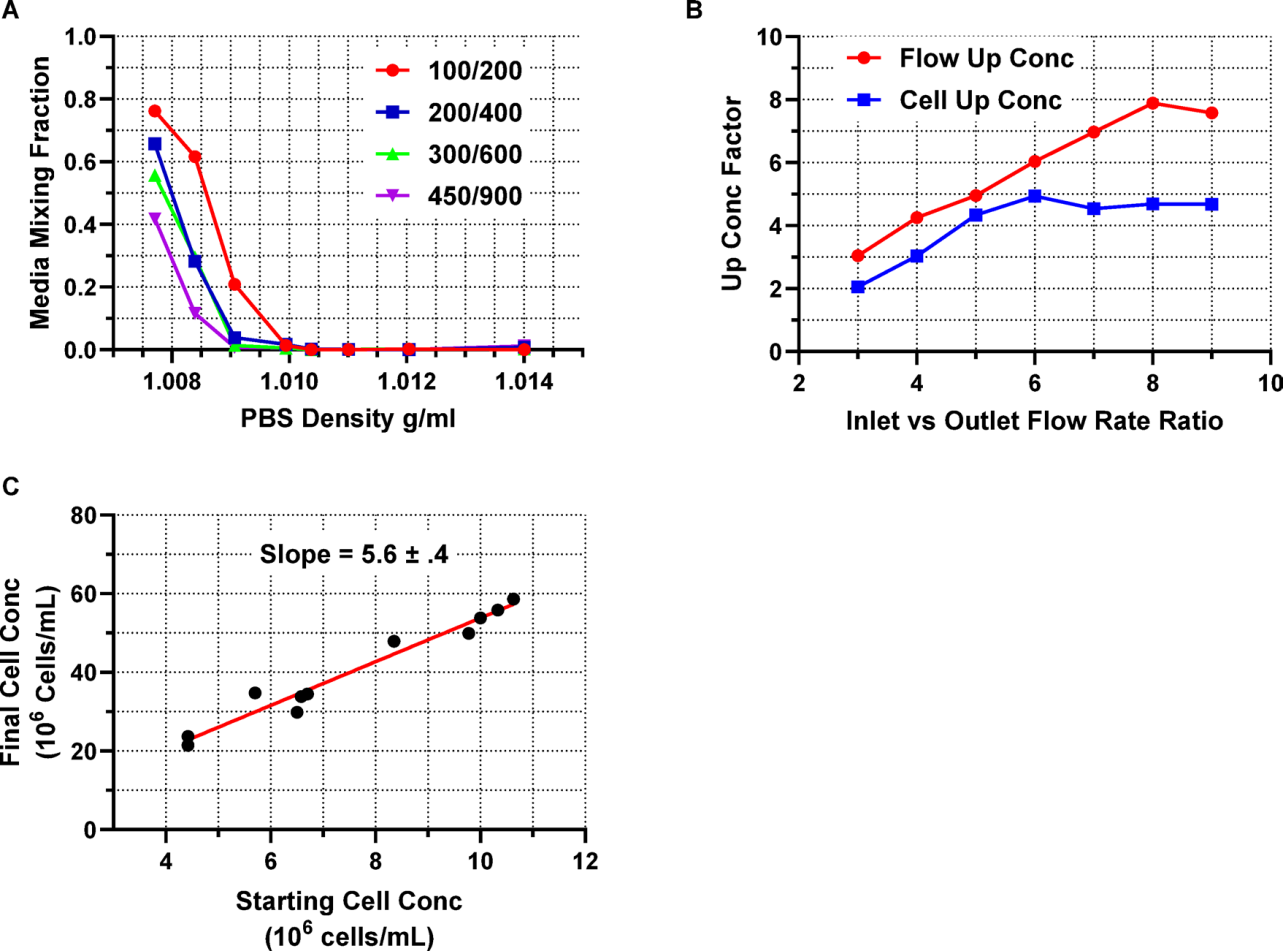
The buffer mixing was affected by the density of the H-PBS used to wash the cells which are suspended in culture medium (**A**). Increasing the flow rate reduced the medium mixing, allowing one to wash cells using a 25:1, or 30:1, PBS to Lymphocyte Separation Medium mixture. The flow rate of 400/800 400/800 was chosen with a PBS to Lymphocyte Separation Medium mixture of 20:1 for all wash cycles. The up-concentration (conc) factor was measured in two ways (**B**). The first was by measuring the volume changes in time and using Eq. 4 (red). The second is by measuring the cell densities before and after the up-concentration wash and using Eq. 3 (blue). The flow rates became unstable at 900 µL/min (red) and the actual up-concentration achieved a maximum at a flow rate of 600 µL/min (blue). From the various radiolabeling tests done during which the radiolabeling procedure was being optimized, the starting and final cell densities were measured for the cell up-concentration wash (**C**). The data shows that an up-concentration factor of 5.6 ± 0.4 was measured independent of the input cell density up to input cell densities of 11 × 10^6^ cells/mL.

From the initial set of tests which characterized the performance of the AcouWash 2, (Fig. 3), we were able to establish the required lymphocyte mixture needed to wash cells suspended in culture medium which is 20 parts PBS to 1 part lymphocyte separation medium with the optimal flow setting of 400/800 400/800 (Fig. 3A). We found the optimal flow rates which maximizes the cell up-concentration to be 600/100 100/600 µL/min, (Fig. 3B), and we established that the up-concentration factor is independent of the initial cell concentration with starting cell concentrations over 10^6^ cells/mL. The mean up-concentrating factor is 5.6 ± 0.4, (Fig. 3C).

### AcouWash 2 cell radiolabeling performance

**Fig. 4:**
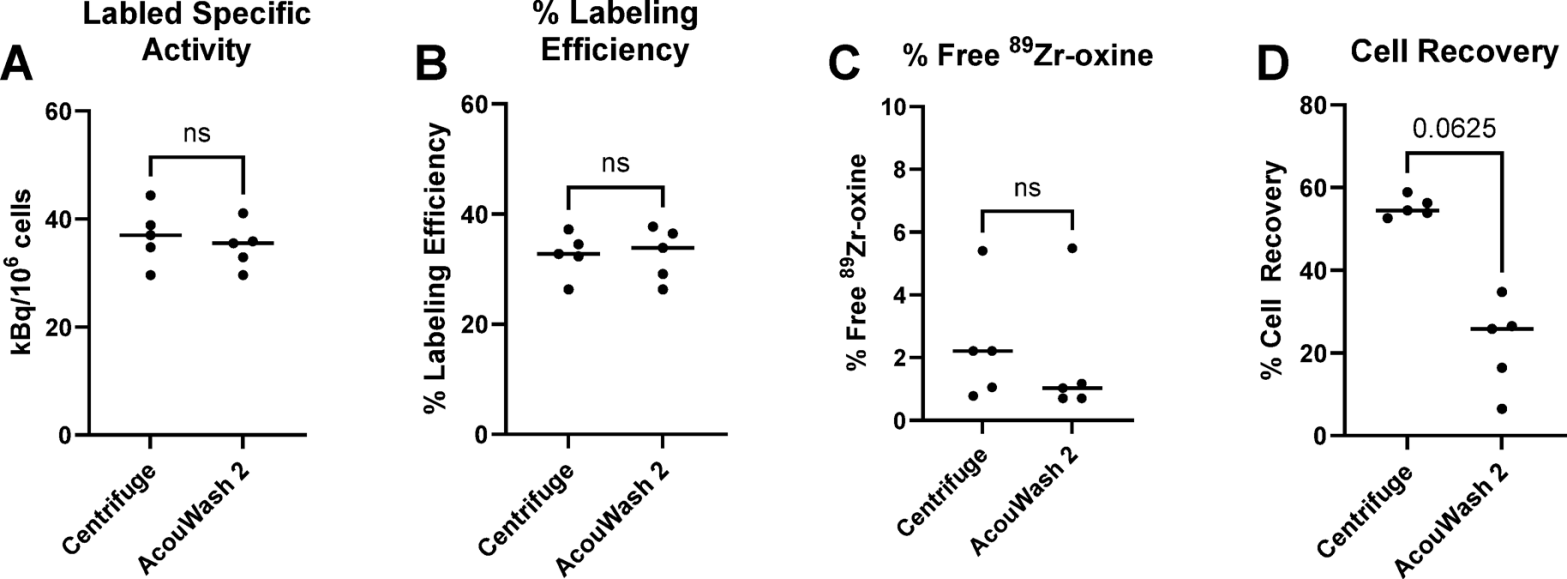
The results of the radiolabeling tests show that the AcouWash 2 radiolabeling metrics are statistically equivalent to the centrifuge ones, except for cell recovery. The results are as follows. The labeled specific activity was 35.9 ± 4.2 kBq/10^6^ cells (AcouWash 2) and 36.9 ± 5.4 kBq/10^6^ cells (centrifuge) (*P* = 0.56) (**A**). The percent labeling efficiency was 32% ± 5% (AcouWash 2) and 32% ± 4% (centrifuge) (*P* > 0.999) (**B**). The percent free ^89^Zr-oxine was 1.8% ± 2% (AcouWash 2) and 2.3% ± 1.8% (centrifuge) (*P* = 0.63) (**C**). The percent cell recovery was 22% ± 10% (AcouWash 2) and 55% ± 2% (centrifuge) (*P* = 0.06) (**D**). *n* = 5. Wilcoxon matched-pairs signed rank test was used for statistical analysis

The radiolabeling procedure was performed five times with the AcouWash 2 and centrifuge procedures done simultaneously, each starting with the same unlabeled cell sample (Fig. 4). The two radiolabeling procedures were done in parallel to minimize any effects due to time differences in the radiolabeling procedure. The resulting radiolabeling performance measurements are as follows: The labeled specific activity was 35.9 ± 4.2 kBq/10^6^ cells (AcouWash 2) versus 36.9 ± 5.4 kBq/10^6^ cells (centrifuge) (*P* = 0.56) (Fig. 4A), the percent labeling efficiency was 32% ± 5% (AcouWash 2) versus 32% ± 4% (centrifuge) (*P* > 0.999) (Fig. 4B), the percent free ^89^Zr-oxine was 1.8% ± 2% (AcouWash 2) versus 2.3% ± 1.8% (centrifuge) (*P* = 0.63) (Fig. 4C) and the percent cell recovery was 22% ± 10% (AcouWash 2) and 55% ± 2% (centrifuge) (*P* = 0.06) (Fig. 4D). Wilcoxon matched-pairs signed rank test was used for statistical analysis.

**Fig. 5:**
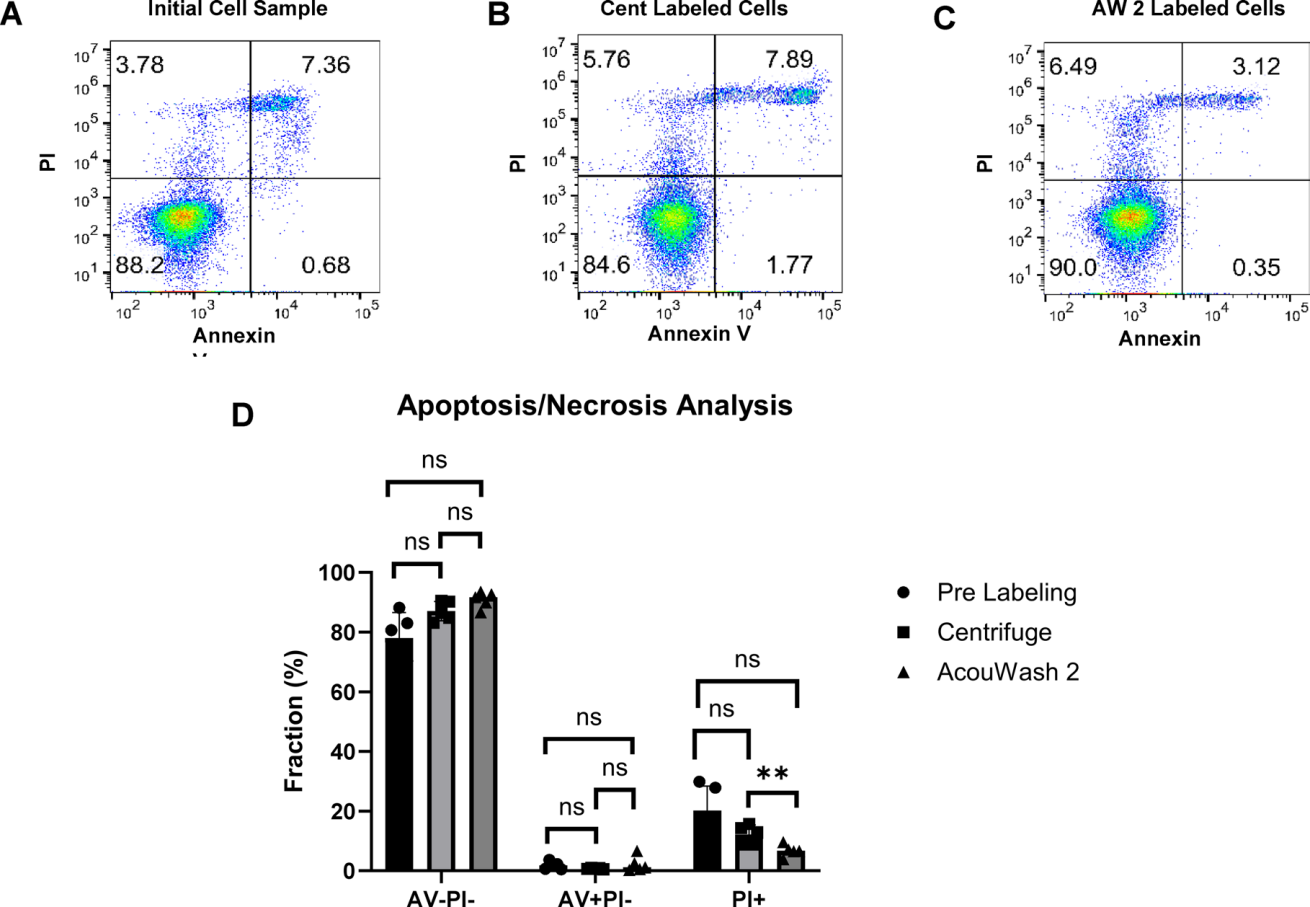
Results of the annexin V and propidium iodide (PI) flow cytometry are shown. Representative scatter plots of annexin V and PI staining are shown for the initial cell sample (**A**), the centrifuge (Cent) radiolabeled cells (**B**) and the AcouWash 2 (AW2) radiolabled cells (**C**) from 5 independent runs. The results were similar among the initial un-radiolabeled cells, the centrifuge and AcouWash 2 radiolabeled cells as summarized in the bar graph (**D**). The annexin V negative and PI negative (AV-PI-) percent fraction was 78% ± 2% (pre labeled), 90% ± 3% (post labeled AcouWash 2) and 87% ± 3% (post labeled centrifuge). The annexin V positive and PI negative (AV + PI-) percent fraction was 1.7% ± 1.3% (pre labeled), 2.2% ± 3% (post labeled AcouWash 2) and 0.8% ± 0.2% (post labeled centrifuge). The positive PI (PI+) percent faction was 20% ± 8% (pre labeled), 7% ± 2% (post labeled AcouWash 2) and 12% ± 3% (post labeled centrifuge). Repeated measure 1-way ANOVA was applied to analyze the effect of the different labeling methods within each stained group individually. All comparisons were not significant (*P* > 0.05) except for the staining group PI + comparing centrifuge to AcouWash 2, which had a *P* = 0.0093

Samples were taken from the initial and final radiolabeled cell suspensions for viability analysis using flow cytometry (Fig. 5). The healthy cell (AV-PI-) percent fractions were 78% ± 2% (pre labeled), 90% ± 3% (post labeled AcouWash 2) and 87% ± 3% (post labeled centrifuge). The apoptotic cell (AV + PI-) percent fractions were 1.7% ± 1.3% (pre labeled), 2.2% ± 3% (post labeled AcouWash 2) and 0.8% ± 0.2% (post labeled centrifuge). The necrotic cell (PI+) percent factions were 20% ± 8% (pre labeled), 7% ± 2% (post labeled AcouWash 2) and 12% ± 3% (post labeled centrifuge). Only the necrotic cell sample comparing the centrifuge to AcouWash 2 radiolabeled cells showed a significant difference with a *P* = 0.0093. This indicates that the necrotic cells of 7% ± 2% contained in the AcouWash 2 radiolabeled samples compared to that of 12% ± 3% contained in the centrifuge radiolabeled samples was statistically significant. All other comparisons were not statistically significant.

### AcouWash 2 cell up-concentration performance

Equation (6) is used to calculate the incubation density. Of the five variables used in the equation, the one that varies most between procedures is the ^89^Zr-oxine specific activity. This is due to the ^89^Zr decay between the time the ^89^Zr-oxine is prepared to the time it is used to radiolabel cells. For this work, the target labeling specific activity was 27.8 kBq/10^6^ cells, the labeling efficiency was 0.3, the PBS to ^89^Zr-oxine volume ratio was 30 parts PBS to 1 part ^89^Zr-oxine and the initial ^89^Zr-oxine specific activity was 74 MBq/mL. Using these parameters in Eq. (6), the incubation density was calculated to be 27 × 10^6^ cells/mL at the time the ^89^Zr-oxine was prepared, which decreases to 14 × 10^6^ cells/mL three days later. For the five radiolabeling test procedures done, the cell concentration for the AcouWash 2 labeling before the concentrating step ranged from 4.4 × 10^6^ cells/mL to 10.3 × 10^6^ cells/mL, requiring concentrating factors ranging from 1.8 to 5.5. The current AcouWash 2 system was able to up concentrate cells by a factor of 5.6 ± 0.4 and so was able to match or exceed the required incubation density for all radiolabeling attempts.

## Discussion

The design of an acoustophoresis-based small cell radiolabeling device would involve compartmentalizing each step in the radiolabeling process within one or more acoustophoresis chips connected in series. The cells would travers the chain of chips, and at each stage the cells would be washed, prepared for incubation or purified preparing them for infusion into a patient.

Before one would commence designing such an automatic radiolabeling system, it is imperative to know if there is anything inherent in microfluidic technology, especially acoustophoresis which would prevent one from radiolabeling cells. Questions need to be answered as to whether the action of the pressure waves caused by the ultrasound transducers affect cell viability. Other questions like whether the labeling efficiency would be compromised, or the purity of the resulting cell suspension can be maintained. The purpose of our work is to answer these questions.

Previously, using the first generation acoustophoresis device (AcouWash, AcouSort, Sweden), we demonstrated that acoustophoresis cell washing can be used to prepare EL4 and mouse T cells for radiolabeling and to wash off the unbound radiotracer after labeling. However, we were unable to achieve the cell concentrations required for the incubation step due to the limited capability of the first generation AcouWash. Thus, a centrifuge was still required for the cell up-concentrating step. With the current version of the device, the AcouWash 2, and its improved system of fluid pumps and monitors, we were able to not just wash cells but also increase their concentration to the levels required for the incubation step without the need for centrifugation.

The final concentration of the cells can be set by adjusting flow settings on the Acouwash 2. The design of the radiolabeling procedure for the AcouWash 2 was such that flow settings were set to achieve maximum up-concentration so that the cells end up in a suspension at a higher concentration than that required by Eq. (6). Then, the cells were diluted down to the required incubation concentration before the ^89^Zr-oxine was added to start the incubation process. This process was used since it gave better control of achieving the incubation concentration set by Eq. (6). It is possible that future iterations will directly deliver the required incubation concentration without the need for dilution.

The radiolabeling test results (Figs. 4 and 5) show that the AcouWash 2 performed as well as the centrifuge method for all test measurements except the cell recovery. The average cell recovery for the AcouWash 2 was 22% ± 10% compared to 55% ± 2% for the centrifuge method. The additional loss of a factor of 2.5 between the AcouWash 2 and the centrifuge is due to the amount of internal tubing in the AcouWash 2. When performing a wash, the wash cycle must be terminated when the suspension volume in the side inlet tube drops to zero, preventing a risk of introducing air bubbles into the tubes, thus leaving cells throughout the inner tubing of the system. Another source of loss is during the up-concentration wash. To maximize the up-concentration factor, a flush is required to ensure the center outlet tubing is full of cells. One then stops the wash, replacing the center outlet tube with an empty one, and continues the wash. There is some loss of cells in the center outlet tube after replacement. Because the goal of this work is to examine if acoustophoresis negatively affects the cell’s viability and their ability to be radiolabeled, the issues regarding cell recovery become less important. In addition, it is expected that once the acoustophoresis radiolabeling procedure is used in a clinical setting, where the number of cells and their suspension volume are increased by at least a factor of 10, the volume of the tubing feeding the side inlet and the center outlet, where the cells are retained after a wash cycle, relative to the total cell suspension will be much smaller thus increasing the cell recovery. Therefore, cell recovery comparisons between the centrifuge and acoustophoresis radiolabeling methods will become more important once a fully automated system has been developed. A standalone radiolabeling system will need to incorporate proper flushing mechanisms and cell management within its design to maximize cell recovery.

Any cell labeling procedure risks damaging cells. Examining the cell viability with flow cytometry, the AcouWash 2 has slightly better cell viability than with the centrifuge. While there is no conclusive explanation for this, the acoustophoresis washing process may have flushed out the necrotic and apoptotic cells through the waste thus increasing the viable fraction of the cell sample. This could become a benefit of acoustophoresis over centrifuge cell labeling methods once a fully automated acoustophoresis radiolabeling system is developed.

This work demonstrates that one can use acoustophoresis technology for each step of the cell radiolabeling process without affecting the cells and achieving the same radiolabeling results as with a centrifuge-based method. Now that this has been demonstrated, the focus of this research will be on designing a fully automated radiolabeling system using acoustophoresis components for the washing and up-concentration steps, with each step feeding the cells into the following step.

## Data Availability

The authors declare that the data supporting the findings of this study are available within the paper. Should any raw data files be needed in another format they are available from the corresponding author upon reasonable request.
